# Case Report: Nintedanib for immune-related pneumonitis triggered by anti-PD-1 treatment in a patient with SMARCA4-mutant NSCLC: a case report

**DOI:** 10.3389/fphar.2023.1177329

**Published:** 2023-05-04

**Authors:** Changwen Deng, Ganxiu Deng, Xiaoping Zhu

**Affiliations:** Department of Respiratory and Critical Care Medicine, Shanghai East Hospital, Tongji University School of Medicine, Tongji University, Shanghai, China

**Keywords:** SMARCA4-mutant, NSCLC, immune checkpoint inhibitors, nintedanib, case report

## Abstract

SMARCA4-mutant lung cancer accounts for approximately 10% of non-small-cell lung cancers (NSCLCs), has few effective treatments, and has been associated with a poor prognosis. Our case report describes a 73-year-old man who was diagnosed with SMARCA4-mutant advanced lung adenocarcinoma. Routine driver gene mutation screening was negative, and tumor tissue immunohistochemistry analysis showed the absence of the BRG1 protein (encoded by SMARCA4). In addition to the standard chemotherapy regimens, programmed cell death protein 1 (PD-1) inhibitors were administered. After three cycles of combination therapy, the focus of the primary lung tumor shrunk evidently, but radiological interstitial abnormalities emerged in the basal and subpleural areas of the bilateral lungs. The patient’s clinical condition deteriorated and he was diagnosed with immune checkpoint inhibitor (ICI)-associated pneumonia. Thus, the combination regimen was discontinued, corticosteroid therapy was administered according to guidelines, and nintedanib was added, given that interstitial abnormalities were observed on chest computed tomography (CT). Following the above treatment, the patient’s condition improved, the standard chemotherapy regimen was restarted, and nintedanib treatment was maintained. The patient’s clinical condition continued to improve, and follow-up CT showed significant resolution of the interstitial abnormalities and stabilization of the primary tumor lesion. In summary, we report the case of a patient with SMARCA4-mutant NSCLC, which is generally considered to be associated with a poor prognosis owing to a lack of effective treatments. The patient responded favorably to initial combination therapy with ICIs, although he subsequently developed immune-related adverse events. We also found that nintedanib, a multitargeted anti-fibrotic agent, was beneficial for the treatment of immune-related lung injury and showed potential anti-tumor effects.

## Introduction

Non-small-cell lung cancer (NSCLC) accounts for 80%–85% of lung cancers and is one of the most common cancers in the world as well as a leading cause of cancer-related deaths (Siegel et al., 2022). Historically, cytotoxic chemotherapy has been the primary treatment option. Owing to the growing depth of cancer biology knowledge and identification of carcinogenic driving genes over the past decade, personalized targeted therapy has become the first-line treatment for patients with NSCLC (Tan and Tan, 2022). However, owing to the lack of targetable gene mutations and presence of drug resistance in some patients, targeted therapy is not suitable for all patients. In recent years, immunotherapy based on immune checkpoint inhibitors (ICIs), including programmed death receptor and ligand 1 (PD-1 and PD-L1) inhibitors, has gradually shifted to first-line therapy and has been found to significantly prolong the survival of patients with advanced NSCLC (Reck et al., 2022). Despite significant improvements, several challenges remain associated with ICI therapy, such as an initial failure response that can be attributed to potential low inherent immunogenicity in certain patients and the development of acquired resistance over time despite an initial promising response (Passaro et al., 2022). Furthermore, increasing evidence has described the acute or chronic clinical toxicities, namely, immune-related adverse events (irAEs), that are associated with the use of these agents.

An increasing number of studies have shown that NSCLCs are associated with cancerous mutations along with non-carcinogenic factors, such as loss of tumor suppressor gene function or abnormalities in the tumor microenvironment (Altorki et al., 2019; Skoulidis and Heymach, 2019). SMARCA4, which encodes the tumor suppressor and transcriptional acting factor BRG1, is a subunit of the switch/sucrose non-fermentable (SWI/SNF) chromatin remodeling complex (Mittal and Roberts, 2020). Through changing the topology of DNA-nucleosomes to regulate gene activity/expression, SMARCA4 is involved in a variety of cellular functions, such as proliferation, differentiation, and DNA repair (Mardinian et al., 2021). SMARCA4 mutations are found in a variety of cancers, including lung cancer, colon adenocarcinoma, bladder urothelial carcinoma, and invasive breast ductal carcinoma, and have been reported in approximately 10% of NSCLCs (Hodges et al., 2018). NSCLC with SMARCA4 deficiency is associated with poor clinical outcomes, and an effective treatment has not been determined (Orvis et al., 2014). Recent studies have suggested that ICIs may show a promising therapeutic response in cancers with SWI/SNF complex mutations (Abou Alaiwi et al., 2020).

## Case presentation

**FIGURE 1:**
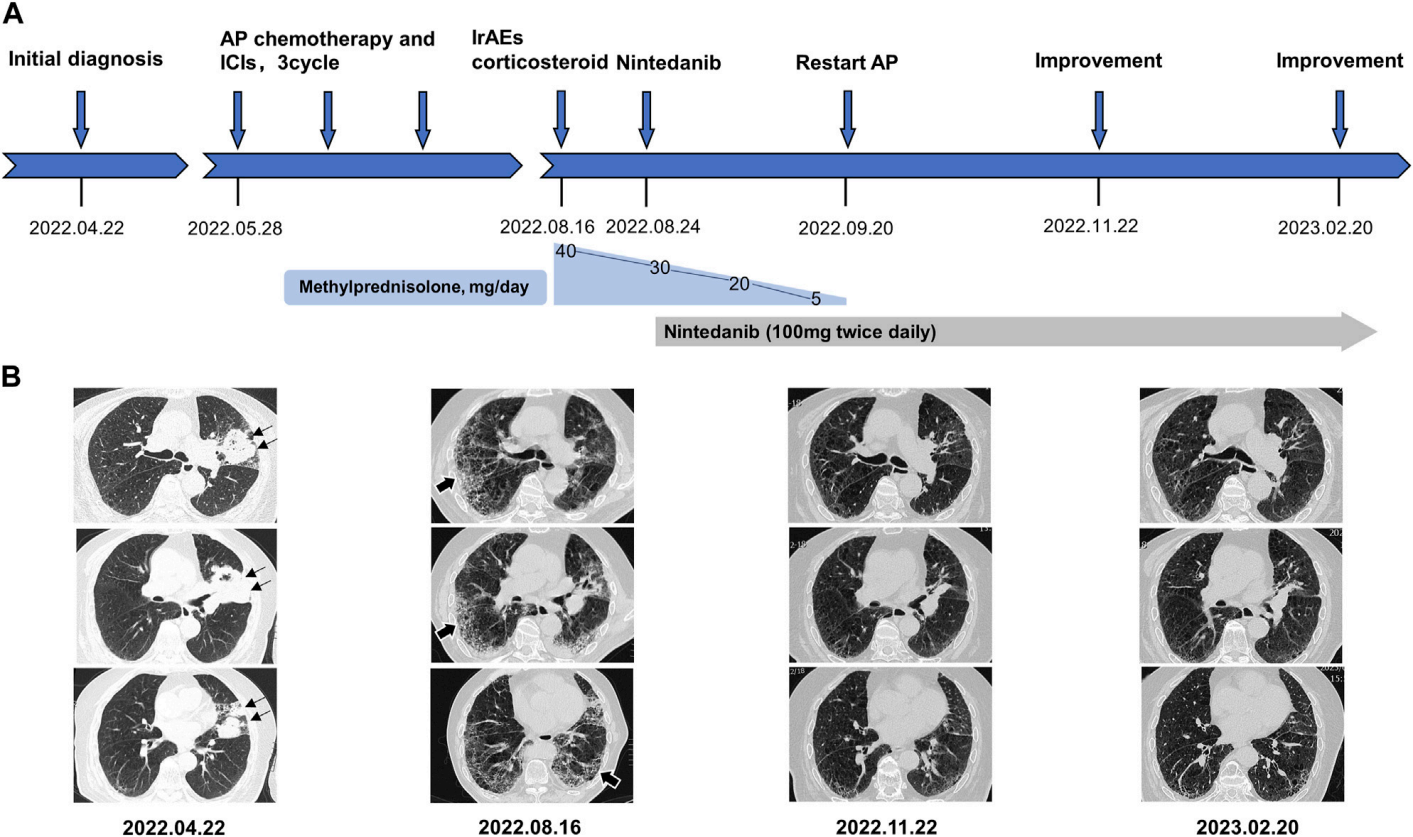
**(A)** refers to the timeline of the patient. **(B)** shows a series of representative different section of chest CT image changes over time. Black thin arrows indicate primary tumor lesion in left upper lung. Black thick arrows indicate immune checkpoint inhibitors-associated pneumonia in bilateral lung. AP chemotherapy, pemetrexed plus carboplatin chemotherapy. irAEs, immune-related adverse events.

A 73-year-old man who presented with a history of blood-stained sputum for 3 weeks was admitted to Shanghai East Hospital with no fever, chest pain, dyspnea, abdominal pain, or nausea. The patient had a smoking history of approximately 50 pack-years. A chest computed tomography (CT) scan in April 2022 showed a mass in the lingual segment of the upper lobe of the left lung, with a cavity and mediastinal lymph node enlargement (Figure 1). On 28 April 2022, the patient underwent endobronchial ultrasound (EBUS) under general anesthesia, and the enlarged lymph nodes were subjected to needle biopsy.

The pathological result of the biopsy specimens indicated poorly differentiated adenocarcinoma, and immunohistochemical staining showed the following: CK(+), CK7(+), TTF1(−), NapsinA(−), BRG1(−), INI1(+), Calretinin(−), WT-1(−), Ki-67(+, 50%), PD-1(lymphocyte +, 10%), PD-L1(polyclonal EIL3N), tumor cell (+)<1%. No driver gene alterations were found in a routine nine-gene mutation test (*EGFR*, *KRAS*, *BRAF*, *HER2*, *NRAS*, *PIK3CA*, *ALK*, *ROS1*, and *RET*). The patient was eventually diagnosed with stage T3N3M0, IIIc left upper lung adenocarcinoma.

**FIGURE 2:**
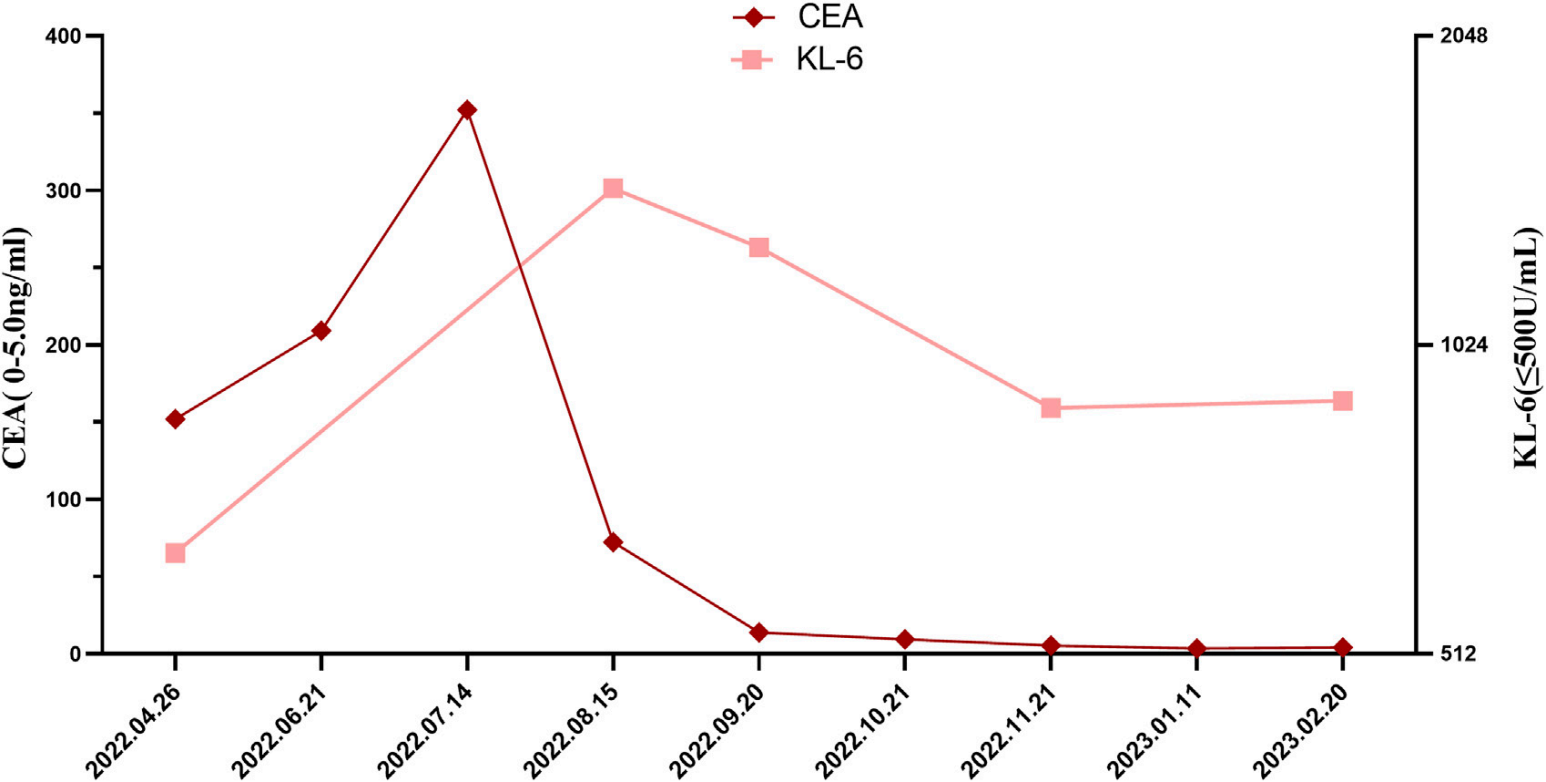
Changes in serum CEA and KL-6 levels through the clinical course. CEA, Carcinoma Embryonic Antigen, KL-6, Krebs von den Lungen-6.

After exclusion of a series of contraindications, the patient began combination therapy with AP chemotherapy (pemetrexed disodium; 500 mg/m^2^, day 1, every 3 weeks) plus carboplatin (AUC 5–6, day 1, every 3 weeks) and camrelizumab (anti-PD-1, 2 mg/kg, every 3 weeks) on 28 May 2022 (Figure 1A). When preparing for the fourth round of combined therapy on 16 August 2022, the chest CT scan suggested a large shrinkage of the primary tumor lesion, but additional interstitial abnormalities in the bilateral basal and subpleural lungs were observed (Figure 1B), along with respiratory distress. The combined treatment was discontinued, oxygen inhalation was initiated, and methylprednisolone (40 mg/day) was added (Figure 1A). The laboratory tests revealed no abnormalities in white blood cell count and C-reactive protein level, but a dramatic elevation of serum tumor markers (carcinoma embryonic antigen; CEA: 72.3 ng/mL [0–5 ng/mL] and Krebs von den Lungen-6; KL-6: 1454 U/mL [0–200 ng/mL]) was observed (Figure 2). The patient was diagnosed with grade 3 camrelizumab-related pneumonitis based on the above findings. Owing to acute dyspnea, bronchoscopy was not performed. After 1 week of corticosteroid therapy, the patient reported no significant symptom alleviation. We then decided to deliver nintedanib (100 mg twice/day), given the fibrosis-like finding on chest CT. After 1 month of corticosteroid treatment combined with nintedanib, the interstitial changes in the chest imaging findings of the patient improved (Figure 1B). Since the tumor treatment needed to be continued, carboplatin plus pemetrexed chemotherapy was restarted on 22 September 2022 and was maintained with nintedanib. During the next 4 months of follow-up, the clinical condition of the patient further improved, with stabilization of the interstitial abnormality and primary tumor lesion (Figure 1) and with significant reductions in serum CEA and KL-6 levels (Figure 2).

## Discussion

The BRG1 protein encoded by SMARCA4, located on chromosome 19q13, is one of two SWI/SNF complex heterodimer catalytic subunits with ATPase activity (Mittal and Roberts, 2020). The SWI/SNF complex consists of three subunits: ATP enzyme catalytic subunit SMARCA4 (BRG1)/SMARCA2 (BRM); highly conserved core subunits SMARCB1 (encoding INI1 protein), SMARCC1, and SMARCC2; and functional specific helper subunits PBRM1 and ARID1A (Mittal and Roberts, 2020). In this patient, the immunohistochemical staining of the tumor tissue showed an absence of BRG1 expression, which indicated *SMARCA4* deletion, and the positive expression of the INI protein was also consistent with the highly conserved characteristics of *SMARCB1*. SMARCA4 has a variety of biological functions that are involved in regulating gene expression, differentiation, and transcription via chromatin remodeling. *SMARCA4* mutations are found in a variety of cancers, including 10% of NSCLCs (Hodges et al., 2018).

In terms of clinical features, most patients with BRG1-deficient NSCLC are smokers (Dagogo-Jack et al., 2020; Schoenfeld et al., 2020; Alessi et al., 2021) and usually diagnosed at an advanced stage. Prior immunohistochemistry (IHC) results have shown that more than 80% of NSCLC tumors can be classified as an adenocarcinoma, although a portion do not express thyroid transcription factor-1 (TTF-1), which is a specific adenocarcinoma marker (Dagogo-Jack et al., 2020). This finding is consistent with the pathological results of our patient. In NSCLCs, *SMARCA4* is rarely co-mutated with certain targeted driver oncogenes, such as *EGFR*, *ALK*, *MET*, *ROS1*, and *RET*, but more frequently co-occurs with other gene mutations, including *KRAS*, *TP53*, *STK11*, and *KEAP1* (Dagogo-Jack et al., 2020; Schoenfeld et al., 2020; Alessi et al., 2021). In our case, a routine nine-oncogene screen was negative, resulting in ineligibility for targeted therapy. The relationship between *SMARCA4* mutations and lung cancer prognosis has attracted increasing attention. A retrospective study of advanced NSCLCs found that patients with the *SMARCA4*-mutation type were associated with shorter overall survival than the wild-type group, and survival was the worst in the BRG1-del group (Schoenfeld et al., 2020; Alessi et al., 2021). Furthermore, patients with BRG1-deficient NSCLCs respond poorly to conventional platinum-based chemotherapy (Bell et al., 2016). This type of cancer is characterized by a lack of targetable mutated genes, insensitivity to chemotherapy, few treatment options, and poor prognosis.

In our case, the patient’s clinical symptoms and primary tumor lesions improved in the early period after receiving combination therapy with ICIs. Several studies have shown that ICI-related immunotherapy can improve the prognosis of patients with *SMARCA4*-mutant NSCLC (Abou Alaiwi et al., 2020; Dagogo-Jack et al., 2020; Schoenfeld et al., 2020). Naito et al. (2019) reported a patient with SMARCA4-deficient NSCLC without targeted driver gene mutations and negative PD-L1 expression who presented a sustained response to nivolumab as a fourth-line treatment. This suggests that ICIs may be a potential promising strategy for such patients, despite their frequent negative expression of PD-L1. Mechanistically, the benefit of this strategy may be related to an elevated tumor burden (Dagogo-Jack et al., 2020; Schoenfeld et al., 2020) and the immune cell infiltration of tumor tissues (Abou Alaiwi et al., 2020). However, other studies have shown no significant correlation between ICIs and improved clinical outcomes in *SMARCA*4-mutant NSCLC (Alessi et al., 2021; Liu et al., 2021). Notably, ICIs showed poor therapeutic effects in these patients, especially in those with *KRAS* co-mutations. The main reasons for the contradictory findings among these studies are that most of the evidence was obtained from retrospective studies and case reports with small sample sizes. The identification of the potential mechanism underlying ICI therapy in *SMARCA4*-mutant NSCLC, such as tumor mutation burden and immune cell infiltration in the tumor microenvironment, may be important for stratifying patients with *SMARCA4*-mutant NSCLC who will benefit from ICIs.

ICIs are revolutionary in the treatment of cancer, especially advanced cancers, and have gradually become the core pillar of cancer therapy. However, with wide clinical application, ICIs have been associated with an increasing number of immune-related adverse events (irAEs) owing to their unique mechanism of action. IrAEs can involve almost all organ systems (Postow et al., 2018), and their onset varies widely, ranging from a few days to several months after administration. Although most of the toxicity is mild and reversible, 0.3%–1.3% of irAEs remain lethal (Postow et al., 2018; Moey et al., 2020), such as immune-associated pneumonia, which is one of the major sources of ICI morbidity and mortality. The most common clinical manifestations are dry cough, dyspnea, and oxygen desaturation. Typical imaging findings include bilateral ground-glass opacities, while other imaging findings include interstitial and organized pneumonia (Wang et al., 2018). More than 85% of patients with pneumonia respond to glucocorticoids and ICI discontinuation, although radiological findings persist for at least 1–2 years in some patients (Johnson et al., 2019).

In this case, the patient developed irAEs approximately 3 months after the initial anti-PD-1 therapy, and chest CT showed bilateral basal and subpleural reticular abnormalities, which improved after treatment with steroids and ICI discontinuation. During the treatment of irAEs, the patients was also administered nintedanib, which was based on the bilateral basement subpleural interstitial changes in CT findings and dramatically increased KL-6 levels.

Nintedanib, an oral multitargeted intracellular tyrosine kinase inhibitor (TKI), has been widely used in the treatment of idiopathic pulmonary fibrosis (IPF). Recently, it has been approved for use in other chronic interstitial lung diseases with fibrotic phenotypes, because it has been found to significantly reduce the annual decline rate of forced vital capacity in multiple well-designed clinical trials (Richeldi et al., 2014; Flaherty et al., 2019; Wells et al., 2020). However, its role in immune-associated pneumonia remains unclear. Fang et al. (2020) found that nintedanib had a significant effect on targeted therapy-related interstitial pneumonia and provided a promising strategy for patients who are not candidates for corticosteroid therapy. Another study reported that nintedanib plus corticosteroids prevented pneumonitis induced by atezolizumab, a PD-L1 inhibitor, in patients with IPF and NSCLC (Yamakawa et al., 2019). Mechanistically, nintedanib can reduce the pulmonary complications of PD-L1 inhibitors and enhance ICI efficacy by promoting vascular normalization, increasing immune cell infiltration and activation in tumors and upregulating MHC-I and PD-L1 expression on the tumor cell surface (Tu et al., 2022). The clinical symptoms in our patient did not improve significantly in the first week after corticosteroid use and ICI withdrawal. When nintedanib was added, the clinical symptoms began to show continuous improvement, and serial chest CT scans showed significant improvement of the pulmonary lesion, which enabled follow-up opportunities to restart anti-tumor treatment.

KL-6 is an extracellular domain epitope of mucins MUC1 and MUC16 and is mainly expressed by alveolar type II epithelial cells and bronchial epithelial cells (Hirasawa et al., 1997). KL-6 is a potential serum biomarker for the diagnosis and prognosis of pulmonary fibrosis (Ohshimo et al., 2014; Hamai et al., 2016). In this patient, KL-6 levels increased dramatically after the development of irAEs, which was associated with interstitial trends, and decreased during nintedanib treatment, which was consistent with radiological improvement.

In addition to its good performance in the treatment of pulmonary fibrosis, nintedanib, originally developed as an anti-neoplastic drug, plays a role in anti-tumor activity because of its multitargeted functions. A multiple phase 3 clinical trial (LUME-Lung 1) in patients with recurrent advanced NSCLC (Reck et al., 2014) revealed that nintedanib combined with docetaxel is an effective second-line treatment, especially for patients with adenocarcinoma. A meta-analysis (Popat et al., 2017) also reported that nintedanib plus docetaxel performed well as second-line treatment and showed better performance in the lower PD-L1 expression group, altogether supporting its clinical use. In our patient, his clinical condition stabilized after restarting chemotherapy and continuing nintedanib, and the primary tumor lesion also stabilized on follow-up CT.

Our study has several limitations. The patient did not undergo testing for *SMARCA4*, despite the nine routine oncogene screening. The *SMARCA4* mutation was identified based on IHC results for the loss of BRG1 expression, although the latter is encoded by *SMARCA4*. In addition to tumor lesions and intestinal abnormalities, the chest CT showed signs of emphysema, which suggested that this patient may have chronic obstructive pulmonary disease (COPD) owing to his long smoking history. However, the patient did not complete lung function tests during the COVID-19 epidemic. Owing to the comorbidities of COPD, the patient was more likely to experience dyspnea at the time of irAEs. This also reminds us of the need for close monitoring in a clinical setting when faced with such patients.

## Conclusion

NSCLC with *SMARCA4* mutations has a poor prognosis and few treatment options. ICI-related immunotherapy may have potential benefits for this type of cancer, but its side effects should be closely monitored. Nintedanib, a anti-fibrotic agent, has shown promising efficacy in the treatment of irAEs and has also been found to have potentially promising anti-tumor effects. The potential synergistic effect of antifibrotic drugs in the treatment of tumors opens up a new therapeutic approach for related complicated diseases with poor prognosis.

## Data Availability

The original contributions presented in the study are included in the article/supplementary material, further inquiries can be directed to the corresponding author.
